# The Effect of Ripening Stages on the Accumulation of Polyphenols and Antioxidant Activity of the Fruit Extracts of *Solanum* Species

**DOI:** 10.3390/plants12142672

**Published:** 2023-07-17

**Authors:** Jūratė Staveckienė, Jurgita Kulaitienė, Dovilė Levickienė, Nijolė Vaitkevičienė, Viktorija Vaštakaitė-Kairienė

**Affiliations:** Department of Plant Biology and Food Sciences, Vytautas Magnus University Agriculture Academy, Donelaičio Str. 58, 44248 Kaunas, Lithuania; jurgita.kulaitiene@vdu.lt (J.K.); dovile.levickiene@vdu.lt (D.L.); nijole.vaitkeviciene@vdu.lt (N.V.); viktorija.vastakaite-kairiene@vdu.lt (V.V.-K.)

**Keywords:** *Solanum* spp. fruits, ripening stage, p-coumaric acid, polyphenols, antioxidant activity

## Abstract

The aim of the research was to evaluate the influence of the ripening stage on the accumulation of polyphenols and antioxidant activity in fruits of *Solanum* species. The experiment included two factors: I—four different *Solanum* species (*S. melanocerasum*, *S. nigrum*, *S. villosum*, *and S. retroflexum*) and II—three ripening stages. High-performance liquid chromatography (HPLC) was used to analyze the individual phenolic compounds (flavonoids and phenolic acids), and the spectrophotometric method was applied to determine antioxidant activity. The results revealed that the accumulation of polyphenols and antioxidant activity in fruits of *Solanum* species depends on the stage of ripening and species. All studied *Solanum* species fruits had the highest content of total phenolic acid at ripening stage III and the greatest antioxidant activity at ripening stage I. Fully ripe fruits of *S. melanocerasum* contained significantly more total flavonoids, whereas *S. nigrum* contained significantly more total phenolic acids than other investigated *Solanum* species fruits. The significantly highest antioxidant activity was found in *S. melanocerasum* fruits at ripening stage I.

## 1. Introduction

The genus *Solanum* and the family *Solanaceae* include several species of edible plants that deserve special attention, also known as wonderberry or sunberry [1,2,3]. The members of the *Solanaceae* family mainly include many species that are widely accepted for their therapeutic properties [4,5,6,7]. One of today’s relevant scientific topics is the search for natural bioactive compounds and their safe and targeted application in the food and pharmacology industries. *Solanum* spp. fruits, due to their rich biochemical composition, are a new and promising research subject in the European and Lithuanian agriculture sectors.

Polyphenols are a diverse group of plant compounds that include flavonoids, phenolic acids, and other related compounds. They are known for their antioxidant properties and potential health benefits [8,9,10,11]. The accumulation of polyphenols in fruits is influenced by various factors, including the ripening stage. Fruit maturity is an important quality factor since it influences both appearance and nutritional value. Polyphenols such as flavonoids and phenolic acids are quality indicators related to maturity. In many fruits, including *Solanum* species, polyphenol content can change during the ripening process [12,13,14]. For example, in some *Solanum* species, such as *Solanum lycopersicum* (tomato), the levels of certain polyphenols, such as flavonoids and phenolic acids, have been reported to increase during fruit ripening. This increase in polyphenol content is often associated with changes in color, flavor, and other characteristics of the fruit [15,16]. However, it is important to note that the specific impact the of ripening stage on polyphenol accumulation can vary among different Solanum species. Furthermore, the growing conditions, environmental factors, and genetic variations can also influence polyphenol levels in fruits [10,13,17].

The ripening stage can also influence the antioxidant activity of *Solanum* species. The ripening process involves complex biochemical changes, including alterations in the levels and composition of various bioactive compounds. These changes can impact the antioxidant activity of *Solanum* fruits. Therefore, in some cases, the antioxidant activity may increase as the fruit ripens, while in other cases, it may decrease or remain relatively stable. The specific changes in antioxidant activity during ripening can vary depending on the Solanum species and the types of antioxidants present [5,7,18]. It is important to consider that the antioxidant activity of Solanum species can be influenced by factors other than the ripening stage. Environmental conditions, cultivation practices, post-harvest handling, and genetic variations among different Solanum species can also affect the antioxidant activity [2].

The main idea of the paper is related to the understudied phytochemical composition and biological activity of the fruits of *S. retroflexum*, *S. melanocerasum*, *S. villosum*, and *S. nigrum*. While the ripening and maturity indications for climacteric fruits are well documented, non-climacteric fruits lag behind [19]. *Solanum* species fruits are among them. In addition, the search for new effective natural active substances with a view to their safe and targeted application in practice is one of the most relevant scientific fields of today. The aim of the research was to evaluate the influence of the ripening stage on the accumulation of polyphenols and antioxidant activity in *Solanum* species.

## 2. Results and Discussion

### 2.1. Flavonoid Content

A diverse group of natural compounds known as flavonoids have been shown to have numerous therapeutic actions against cancer, neurological diseases, cardiovascular diseases, diabetes, inflammation, and many other diseases [20].

**Figure 1:**
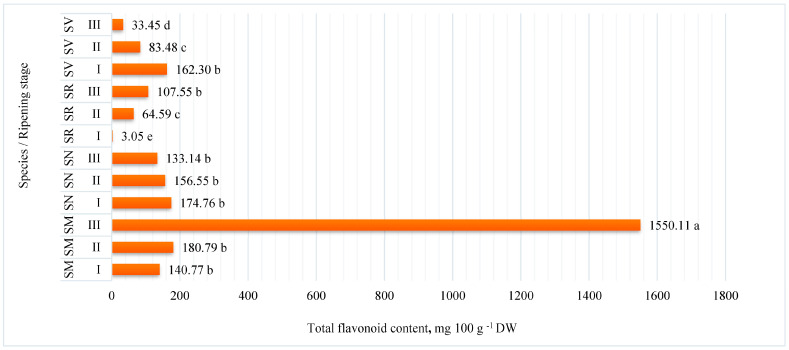
Total flavonoid content (mg 100 g^−1^ DW) in *Solanum* fruits at different ripening stages. Note: Different small letters (a–e) represent significant differences between the means (*p* < 0.05); *S. retroflexum—*(SR), *S. melanocerasum*—(SM), *S. nigrum*—(SN), *S. villosum*—(SV); Ripening stages I, II, III.

The flavonoid contents were quantified in *S. retroflexum*, *S. melanocerasum*, *S. nigrum*, and *S. villosum* species at different ripening stages by HPLC-DAD. The data averaged over the two experimental years are shown in Figure 1. The total amounts of flavonoids in *Solanum* fruits significantly depend on species and ripening stage. *S. melanocerasum* fruits showed significantly the highest amounts of total flavonoids (1550.11 mg 100 g^−1^ DW) at ripening stage III. In addition, the other species of *Solanum* fruits showed lower amounts of total flavonoid content at all ripening stages, and the significantly lowest was 3.05 mg 100 g^−1^ DW in *S. retroflexum* fruits at ripening stage I. 

Kamau et al. [19], investigated the content of total flavonoids in fruits of four *S. nigrum* cultivars harvested at four stages (green, color break, ripe, and senescence). The highest content of this compound was found in the ‘Giant Nightshade’ and ‘JKUAT’ cultivars (455 mg 100 g^−1^ and 502 mg 100 g^−1^, respectively) when the fruits were ripe. However, total flavonoid content in the cultivars ‘Black Nightshade’ and ‘KARLO’ increased as the fruits started breaking their colors and then diminished through the last stages of maturity. Dūma et al. [21], studied the influence of variety and stage of maturity on the contents of bioactive compounds in fruits of *Solanum lycopersicum* L. (tomato). They found that the content of total phenols and total flavonoids increased during tomato ripening.

In *Solanum,* fruits were quantified by five flavonoids, including epicatechin, rutin, myricetin, quercetin, and apigenin, which are shown in Table 1. Variations among individual flavonoids were investigated according to the ripening stage in all four species. The data demonstrated that the significantly highest concentration of flavonoid rutin was detected in *S. melanocerasum* species when the fruits were fully ripe. Depending on the species, the rutin contents in *Solanum* fruits fluctuated from 0.48 mg 100 g^−1^ to 1448.30 mg 100 g^−1^ DW. The lowest amount of rutin was established at ripening stage I in *S. retroflexum* fruits (Table 1).

Epicatechin was another important flavonoid found in *Solanum* fruit. In addition to its direct antioxidant effect, epicatechin in small doses can protect other antioxidants such as vitamins C and E [22]. Results in Table 1 show that the content of epicatechin in *Solanum* fruits ranged from 0.85 mg 100 g^−1^ at ripening stage I in *S. retroflexum* species, to 117.05 mg 100 g^−1^ DW at ripening stage II in *S. melanocerasum*. As reported by Chang et al. [23], the main flavonoids detected in *S. nigrum* fruit extract were rutin and epicatechin. Results by Elmastas et al. [24] showed that the content of epicatechin in *Rosa* species ranged from 225.25 to 472.67 mg kg^−1^. According to these authors, the content of this compound depends on the ripening stage and increases during the growth period. The significantly highest content of myricetin (9.54 mg 100 g^−1^) was determined in *S. melanocerasum* fruits at the beginning of the experiment. However, the ripening stage had no significant effect on the content of this compound in the fruits of *S. retroflexum* and *S. villosum*. The content of quercetin and apigenin also varied widely depending on the species and ripening stage. In the fruits of *S. melanocerasum*, *S. nigrum*, and *S. villosum* the significantly highest contents of this flavonoid were established at ripening stage I (14.38 mg 100 g^−1^, 15.15 mg 100 g^−1^, and 9.98 mg 100 g^−1^, respectively), while in *S. retroflexum* the highest contents were identified at ripening stages II and III. Significantly the highest content of apigenin was detected at ripening stage II, 26.61 mg 100 g^−1^ DW in *S. villosum* fruits. The significantly lowest contents of this compound were found in the fruits of *S. retroflexum*, *S. nigrum,* and *S. villosum* at ripening stage I, while in *S. melanocerasum* the lowest contentwas found at ripening stage III.

According to the literature, the variation of flavonoids can depend on several factors, including genetic variations, environmental conditions, and the maturity of the fruit. Cultivation practices and growing conditions can also influence the flavonoid content of *Solanum* fruits [25]. 

### 2.2. Phenolic Acid Content 

**Figure 2:**
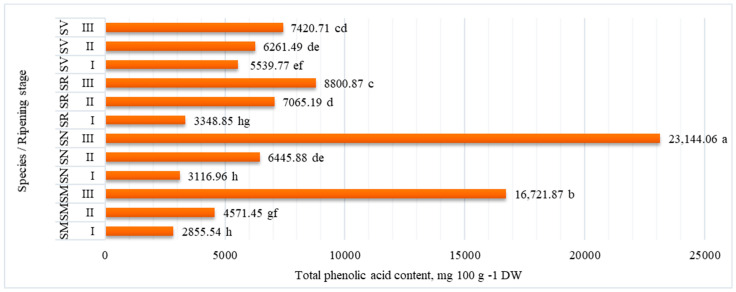
Total phenolic acid content (mg 100 g^−1^ DW) in *Solanum* fruits at different ripening stages. Note: Different small letters (a–h) represent significant differences between the means (*p* < 0.05); *S. retroflexum*—(SR), *S. melanocerasum*—(SM), *S. nigrum*—(SN), *S. villosum*—(SV); Ripening stages I, II, III.

Phenolic acids are a group of secondary metabolites found in plants, including *Solanum* species. The total phenolic acid content of *Solanum* species fruits are presented in Figure 2. All *Solanum* species were produced under identical conditions, but the amount of phenolic acid accumulation in the fruits varied greatly depending on the stage of ripening and species. 

The amount of total phenolic acid was found to range from 2855.54 mg 100 g^−1^ DW (at ripening stage I in *S. melanocerasum* fruit) to 23,144.06 mg 100 g^−1^ DW (at ripening stage III in *S. nigrum* fruits), with the greatest values observed at the fully ripe stage (ripening stage III). The fruits of all the studied *Solanum* species had the lowest content of total phenolic acid at ripening stage I (fruits color green).

Eight phenolic acids, including p-coumaric acid, chicoric acid, ferulic acid, m-coumaric acid, o-coumaric acid, rosmarinic acid, protocatechuic acid, and chlorogenic acid were investigated in all four species (Table 2). Our results revealed that the contents of individual phenolic acids vary widely depending on the ripening stage. Furthermore, the different *Solanum* species revealed different trends regarding content of these phenolic acids.

Almost all the phenolic acids (except protocatechuic acid) in *S. melanocerasum* fruits increased during the ripening process. The fruits of this species showed the highest contents of chicorc, ferulic, m-coumaric, and rosmarinic acids at ripening stage III (9260.75 mg 100 g^−1^, 2472.87 mg 100 g^−1^, 2771.45 mg 100 g^−1^ and 79.49 mg 100 g^−1^, respectively), as compared to other examined species‘ fruits. Similarly, the contents of p-cumaric acid, chicorc, ferulic, and protocatechuic acids (12,813.79 mg 100 g^−1^, 3244.17 mg 100 g^−1^, 566.60 mg 100 g^−1^, and 6040.11 mg 100 g^−1^, respectively) at ripening stage III in *S. nigrum* fruit were significantly higher than those at ripening stages I or II. However, in *S. nigrum* fruits, the significantly highest contents of o-coumaric and m-coumaric acids were found at ripening stage II, while the highest contents of rosmarinic and chlorogenic acids were found at ripening stage I. Our data showed that the contents of individual phenolic acids in *S. retroflexum* fruits varied greatly depending on the ripening stage. This species of fruits had the highest content of chicoric acid at the fully ripe stage (ripening stage III), ferulic and protocatechuic acids at ripening stages II and III, p-cumaric acid at ripening stage I, and chlorogenic acid at ripening stage II. However, the ripening stage had no significant effect on the contents of p-cumaric and m-cumaric acids in the fruits of *S. retroflexum*. *S. villosum* showed significantly the highest contents of p-cumaric, chicorc, ferulic, and m-cumaric acids at ripening stages II and III. The ripening stage had no significant effect on the contents of o-coumaric and rosmarinic acids in the fruits of this species.

Xie Goufang et al. [26] determined that the main phenolic compounds in rabbiteye blueberries during ripening were ferulic acid, rutin, epicatechin, quercetin, chlorogenic acid, catechin, and p-coumaric acid. The contents of these compounds ranged from 0.27 to 128.96 mg·kg^−1^, 0.44 to 67.40 mg·kg^−1^, 0.59 to 66.49 mg·kg^−1^, 0.46 to 36.79 mg·kg^−1^, 0.39 to 24.59 mg·kg^−1^, 1.30 to 11.55 mg·kg^−1^, and 0.01 to 7.58 mg·kg^−1^, respectively. Results showed that the contents of phenolic acids increased during ripening. 

The study by N’Dri et al. [27], investigated individual phenolic acids in different ripening stages (fruit colors green, yellow, and red) of fruits of *Solanum indicum* L. Almost all the phenolic acids (particularly caffeoylquinic and coumaroylquinic acids) in fruits increased as they ripened. The contents of quercetin-3-O-rutinoside, quercetin-3-O-glucoside, and kaempferol-3-O-rutinoside in red fruits were about 10 times higher than those of green and yellow fruits. However, ferulic and p-coumaric acids content remained stable throughout the ripening process.

These studies suggest that the ripening stage plays a role in the accumulation of phenolic acids in fruits. However, it is important to note that the specific changes in phenolic acid content during ripening may vary depending on the particular phenolic acid compounds and their concentrations and factors such as growing conditions, cultivar differences, and analytical techniques used for quantification [10,13,28].

### 2.3. Antioxidant Activity of Fruit Extracts

**Figure 3:**
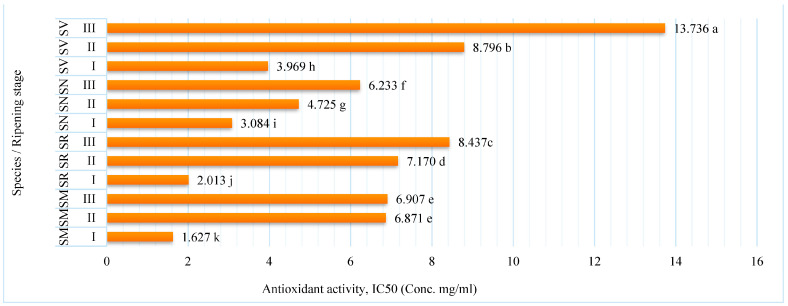
Antioxidant activity (IC_50_) of *Solanum* fruit extracts at different ripening stages. Note: Different small letters (a–k) represent significant differences between the means (*p* < 0.05); *S. retroflexum*—(SR), *S. melanocerasum*—(SM), *S. nigrum*—(SN), *S. villosum*—(SV); Ripening stages I, II, III.

Antioxidant activity is an important property of many plant compounds, including polyphenols, which are often associated with antioxidant capacity. The antioxidant activity of a food product, which assesses its ability to prevent oxidation, is an important component in evaluating its health benefits. Since oxidation is a key to many human diseases and the aging process, it is a desirable characteristic of food [29]. Due to the presence of numerous natural antioxidants such as phenolic compounds and flavonoids, *Solanum* fruits have high antioxidant qualities. The scavenging effects of various sample extracts of antioxidant activity were presented in Figure 3. The results of the experiment demonstrated that the lower the IC50, the greater the antioxidant activity of fruit extracts [30].

The specific changes in the antioxidant activity of fruit extracts during ripening can vary depending on the *Solanum* species. As presented in Figure 3, in all *Solanum* fruits, antioxidant activity varied from 1.627 IC50 SM for fruits at ripening stage I to 13.736 IC50 SV for fruits at ripening stage III. The antioxidant activity decreased as the fruit ripened. The significantly highest antioxidant activity in the fruit extracts was determined at ripening stage I in all investigated species. While at ripening stage III, all species demonstrated significantly lower amounts of this activity. SM fruits had significantly greater antioxidant activity compared with other species at all stages of ripening.

According to Cano et al. [31], the total antioxidant activity of fruit is highly reliant on the ripening stage. The total antioxidant activity decreased during ripening, and this increase is mostly caused by lipophilic antioxidant activity changes. 

Bhandari S.R and Lee J.G. [32], stated that antioxidant activities in different maturity stages were cultivar-dependent. They found, that the higher activities were observed in cherry tomato varieties (DPPH: 1596–2552 μmol TE/100 g) than in general varieties (DPPH: 579–1627 μmol TE/100 g). Furthermore, antioxidant activity varied significantly between ripening stages; antioxidant activity increased from the breaker stage to the red stage. This is confirmed by research by Periago et al. [33]. These scientists reported that antioxidant activity increased significantly during ripening in extracts of all three different cultivars of tomato. 

Fattahi et al. [34] investigated the influence of different species and ripening stages on the antioxidant activity of grapes. The results showed that the higher antioxidant activity was in green grapes, and it varied from 0.25 to 11.54 IC50. 

There is little information about how the ripening stage of fruits affects their bioactive ingredients, their antioxidant capabilities, and how these components relate to total antioxidant activity. In this study, results indicated the different effects of the ripening stage on the antioxidant capacity of *Solanum* fruits.

### 2.4. Principal Component Analysis (PCA)

**Figure 4:**
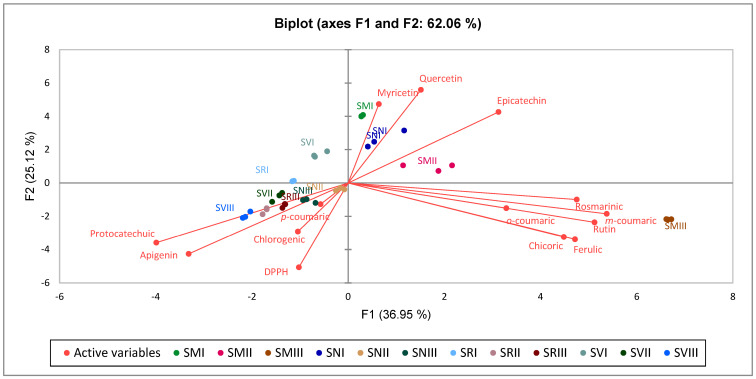
Principal component analysis of investigated compounds in *Solanum* species fruits. Note: *S. retroflexum*—(SR), *S. melanocerasum*—(SM), *S. nigrum*—(SN), *S. villosum*—(SV); Ripening stages I, II, III.

The PCA biplot shows relationships between the average content of individual phenolic compounds and DPPH radical scavenging activity in fruits of various *Solanum* species at different ripening stages (Figure 4).

In general, the PCA biplots showed distinct results of SMIII to other *Solanum* spp. species at different ripening stages. SMI, SMII, and SN I showed distinct results to SN II-III, SRIII, SV II-II, and SM III. SV I and SR I was not associated with any phenolic compound or DPPH radical scavenging activity.

**Figure 5:**
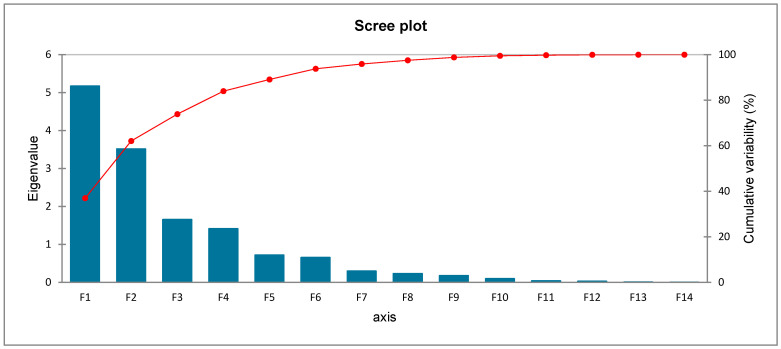
Scree plots of principal component analysis.

The scree plots of the PCA showed that the first two eigenvalues accounted for most of the variance in the data (Figure 5).

The PCA factor loadings, scores, and eigenvalues for the first two principal components (F1 and F2) are presented in Table 3. Factor loadings, eigenvalue, variability (%), cumulative variability (%), and score for the first two principal (F1–F2) components for individual phenolic compounds and DPPH radical scavenging activity in fruits of various Solanum species at different ripening stages are shown.

The first two PCAs extracted from the components amounted to 62.06% of the total data. The PCA indicates that epicatechin, myricetin, and quercetin were associated with SM I-II and SN at ripening stage II, which had a positive score along with F1 and F2. Rosmarinic acid, m-coumaric, o-coumaric, rutin, ferulic acid, and chicoric acid were associated with SM at ripening stage III, which had a high positive score along F1 and negative along with F2. Chlorogenic acid, p-coumaric, apigenin, protocatechuic acid, and DPPH radical scavenging activity were associated with SV at ripening stage II, SN at ripening stages II and III, and SR and SV at ripening stage III, which had negative score along F1 and F2. There were no associations between F1 and F2 scores of the SV and SR at ripening stage I with individual phenolic compounds and DPPH radical scavenging activity.

## 3. Materials and Methods

### 3.1. Field Experiment

**Figure 6:**
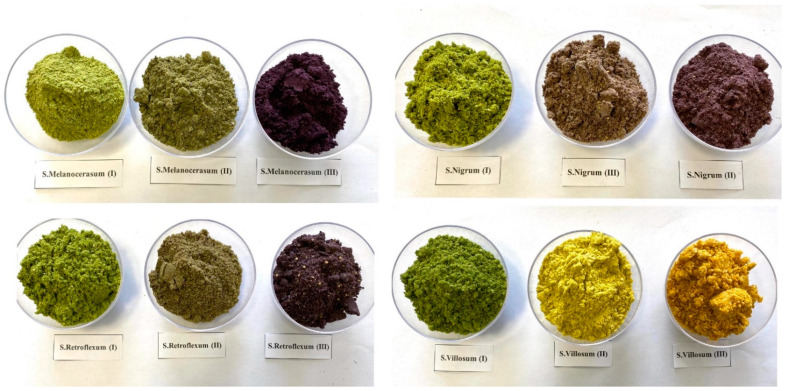
Fruit ripening stages of *Solanum* spp. represented by milled fruit samples (photos by J. Staveckienė).

A two-factor experiment with Solanum species: (SM)—*S. retroflexum*, (SN)—*S. nigrum*, (SV)—*S. villosum*, and (SM)—*S. melanocerasum*, and three different ripening stages: I—ripening stage, fruit color green (30% maturity); ripening stage II, fruit color 40–60% purplish-violet or yellow-orange (60% maturity), inside incompletely ripe; and III−ripening stage, fruit color 100% velvety black-blue or orange, inside fully ripe (100% maturity) (Figure 6) [35,36] was conducted during the period 2020–2021 on a farm in the Kaunas district, Lithuania (WGS coordinates 54.8719020, 23.8672686).

The seedlings were inserted into the soil for the field experiment. The strongest seedlings were transplanted to the field in the third ten-day period of May after the seeds were seeded in nurseries in March. Before planting, a black synthetic film was placed over the soil’s surface; holes were then drilled through the film, and the seedlings were inserted there. Under the agro-film, a drip irrigation system was set up, with the watering rate set at 1 L per hour as necessary for the weather. Four replicates of each treatment were used in the experimental plots, which were organized in randomized blocks. Four seedlings of each species were included in each replication. Each experimental area was 1.5 m broad and 7.5 m long. 

The protective zone and the complete experimental area were 148 m^2^. Between July 1 and the first frost, fruits were systematically collected at all three ripening stages. The fruit ripening stages, which were visually examined, and the weather conditions affected each experimental year’s harvest dates.

### 3.2. Sample Preparation

After collecting the fruits, for analysis, a combined sample was made of 1.5 kg for each species and each stage of ripening. The fruits were cleaned with tap water, dried, and kept at −34 °C. The samples were lyophilized for 24 h using a Freeze–Drying Plant Sublimator 304 05 (ZIRBUS Technology GmbH, Bad Grund, Germany). Following lyophilization, the fruits were ground (Grindomix GM 200, Retsch GmbH, Haan, Germany) and kept at 5 °C in the dark before being subjected to a polyphenols and antioxidant activity analysis. 

### 3.3. Soil Agrochemical Analyses

An agrochemical auger was used to collect soil samples from the arable layer (0–20 cm depth) in the spring. The soil samples were homogenized, sieved through a 1 mm mesh sieve, and air dried in transparent plastic cases. Agrochemical analyses of the experimental soil were conducted at the Laboratory of Analyses of Vytautas Magnus University Agriculture Academy. The pH, KCl, phosphorus, potassium, and total nitrogen concentrations of soil samples were analyzed. Soil pH was measured according to the potentiometric method using a pH meter in 1 N KCl extract (Lithuanian organization LST) [37]. Using the Egner–Riehm–Domingo method, the ammonium-lactate extraction of the available phosphorus and potassium was performed [38]. The total nitrogen concentration (mg kg^−1^) was determined using the Kjeldahl method. The experimental field soil was characterized by acidity (pH = 4.16), medium potassium status (K_2_O = 78.5–102.2 mg kg^−1^), low phosphorus status (P_2_O_5_ = 45.9–69.3 mg kg^−1^), and 1.25% total nitrogen content.

### 3.4. Meteorological Conditions

The weather data during the experimental period in 2021 and 2022 are shown in Table 4. During the years 2021 and 2022, the climate was warmer (by 0.58 and 0.42 °C, respectively) in comparison with the standard climate normal (SCN). The rainfall in 2021 and 2022 during the vegetation period was lower. In 2021 the sum of rainfall was 391.50 mm and in 2022 was 344.50 mm, in comparison with the SCN value of 395 mm. 

### 3.5. Determination of Polyphenolic Compounds 

Individual phenolic compounds were analyzed by high-performance liquid chromatography (HPLC) according to Brazaitytė et al. [39]. 

**Figure 7:**
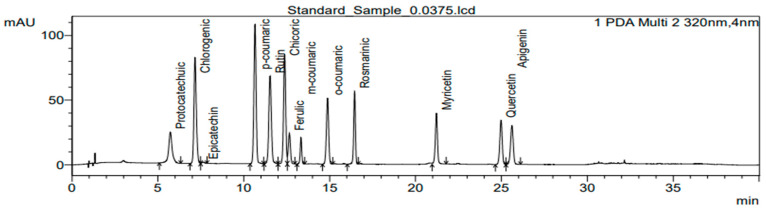
Chromatogram of standards retention time.

For the extraction of phenolic compounds, 100 mg of lyophilized plant material was ground with 80% methanol (Sigma-Aldrich, St. Louis, MO, USA) and transferred to a 15 mL polypropylene conical centrifuge tube (Labbox Labware S.L., Barcelona, Spain). The extract was incubated at 4 °C for 24 h. Samples were filtrated through a 13 mm and 0.22 µm nylon syringe filter (BGB Analytik AG, Böckten, Switzerland). The UFLC 10A system (Shimadzu Nexera X2, Kyoto, Japan) equipped with a diode array (SPD-M30A) detector was used for analysis. The sample was separated on a Triart C18 plus column (3 µm particle size, 100 × 3.0 mm) (YMC Europe GmbH, Dinslaken, Germany). Peaks were detected at 320 nm. The mobile phase consisted of A (100% acetonitrile, (Sigma-Aldrich, St. Louis, MO, USA) and B (1% acetic acid, Supelco, Bellefonte, PA, USA). Binary gradient: 0 min; 95% B, 25 min; 70% B, 25–30 min; 5% B, 30–35; 5% B; 35–37 min; 95% B, and 37–40 min; 95% B, flow rate 1 mL min^−1^. The results are expressed as an average of analytical measurements of three biological samples from homogenized plant material in mg g^−1^ in the dry mass of plants. The contents of rutin (rutin trihydrate, Supelco), myricetin, chicoric acid, ferulic acid (trans-Ferulic acid), rosmarinic acid, quercetin, and protocatechuic acid (all purchased from Merck KGaA, Darmstadt, Germany), caffeic acid, p-coumaric acid (trans-p-Coumaric acid), o-coumaric acid (trans-2-Hydroxycinnamic acid), m-coumaric acid (trans-3-Hydroxycinnamic acid), epicatechin, chlorogenic acid, kaempferol, gallic acid (gallic acid monohydrate) (all purchased from Sigma-Aldric), and apigenin (LGC Standards Ltd., LGC, Teddington, UK) are expressed as mg g^−1^ in the dry matter of plants (Figure 7). 

The total amount of phenolic acids and flavonoids was determined by summing up individual compounds made according to the methodology already described.

**Figure 8:**
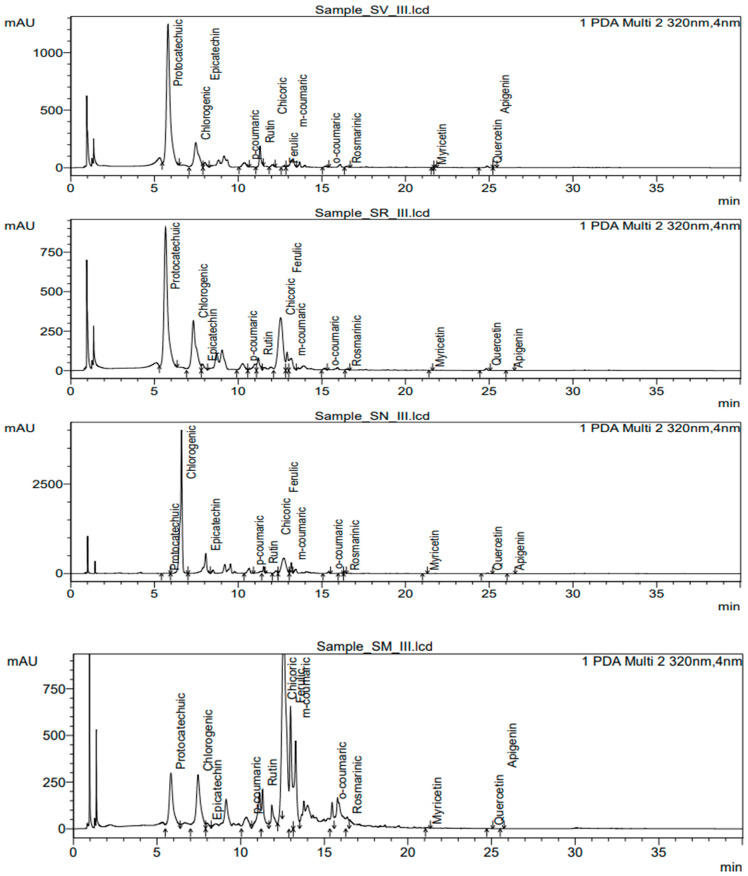
Individual polyphenolic compounds chromatograms in SV III*S. villosum* ripening stage III, SR III—*S. retroflexum* ripening stage III, SN III—*S. nigrum* ripening stage III, SM III—*S. melanocerasum* ripening stage III.

During the determination of polyphenol compounds in a sample, the retention time of compounds can shift as high as 0.5 min. It depends on the concentration of the compound and sample preparation. In Figure 8, we present chromatograms of the third ripening stage for each species.

### 3.6. Determination of DPPH Radical Scavenging Activity

The DPPH free radical scavenging activity of each sample was determined using the Ultraspec 3000 UV/vis Spectrophotometer (Pharmacia Biotech Ltd., Cambridge CB44FJ, UK) according to the method described by Leong and Shui [40]. In a nutshell, methanol was used to prepare a 0.1 mM solution of DPPH. At 515 nm, the initial DPPH in methanol absorbance was recorded, and it remained constant during the course of the test. An aliquot of 0.4 mL of an extract was added to 3 mL of methanolic DPPH solution. The change in absorbance at 515 nm was measured at 30 min.

Results have also been reported as IC50, which is the amount of antioxidants necessary to decrease the initial DPPH concentration by 50%. All the tests were performed in triplicate. 

### 3.7. Statistical Analysis

Statistical analysis was performed using Microsoft Excel 2016 and Addinsoft XLSTAT 2022 XLSTAT statistical and data analysis (Long Island, NY, USA). A two-way analysis of variance (ANOVA) followed by Tukey’s significant difference test (*p* < 0.05) for multiple comparisons was used to evaluate differences between means (*n* = 3) of measurements.

## 4. Conclusions 

Overall, the results of this study demonstrate that the highest content of total phenolic acid content occurred at ripening stage III in *S. nigrum* fruits (23,144.06 mg 100 g^−1^ DW). The qualitative and quantitative composition of flavonoids also depends on the ripening stage. *S. melanocerasum* fruits showed significantly the highest amounts of total flavonoids (1550.11 mg 100 g^−1^ DW) at ripening stage III. Antioxidant activity decreased as the fruit ripens. Significantly highest antioxidant activity was determined at ripening stage I in all investigated species.

Our study confirms that the fruits of the investigated *Solanum* species are a good source of polyphenols. These data are valuable for choosing a ripening stage with the highest accumulation of phenolic acids and flavonoids in fruits.

Therefore, fruits from *Solanum* species are encouraged for use in food, cosmetics, and pharmaceuticals. To understand the process relating to the effect of the fruit ripening stage on quality, further investigations are required.

## Figures and Tables

**Table 1:** Individual flavonoid content (mg 100 g^−1^ DW) in *Solanum* fruits at different ripening stages.

Species	Ripening Stage	Epicatechin	Rutin	Myricetin	Quercetin	Apigenin
**SM**	I	99.54 bc	14.86 d	9.54 a	14.38 a	2.46 f
**SM**	II	117.05 b	52.22 b	4.02 b	6.18 c	1.32 fg
**SM**	III	92.03 c	1448.30 a	3.08 bcd	5.83 c	0.88 g
**SN**	I	146.35 a	8.87 d	3.48 bc	15.15 a	0.91 g
**SN**	II	91.94 c	47.49 b	1.80 cd	2.34 de	13.00 d
**SN**	III	69.32 d	48.48 b	3.850 b	0.44 f	11.32 e
**SR**	I	0.85 f	0.48 e	1.26 d	0.22 f	0.23 g
**SR**	II	7.13 f	26.24 c	2.97 bcd	3.73 cde	24.52 b
**SR**	III	51.63 de	30.30 c	2.60 bcd	1.29 de	21.73 c
**SV**	I	94.65 c	52.95 b	4.19 b	9.98 b	0.53 g
**SV**	II	42.32 e	6.88 d	3.17 bc	4.50 cd	26.61 a
**SV**	III	2.26 f	0.71 e	2.92 bcd	3.66 cde	23.90 b
**P > F (Model)**		<0.0001	<0.0001	<0.0001	<0.0001	<0.0001
**Significant**		Yes	Yes	Yes	Yes	Yes

Note: Different small letters (a–g) at the same column represent significant differences between the means (*p* < 0.05); *S. retroflexum*—(SR), *S. melanocerasum*—(SM), *S. nigrum*—(SN), *S. villosum*—(SV); Ripening stages I, II, III.

**Table 2:** Individual phenolic acid content (mg 100 g^−1^ DW) in *Solanum* fruits at different ripening stages.

Species	Ripening Stage	P-Cumaric Acid	CHICORIC ACID	Ferulic Acid	M-Coumaric Acid	O-Coumaric Acid	Rosmarinic Acid	Protocatechuic Acid	Chlorogenic Acid
**SM**	I	11.026 d	390.05 e	321.69 de	296.60 c	6.72 e	2.77 g	1647.94 c	178.75 g
**SM**	II	22.952 d	632.62 d	1103.89 b	1018.20 b	47.43 c	39.21 bc	1375.98 c	331.17 f
**SM**	III	243.64 b	9260.75 a	2472.87 a	2771.45 a	66.37 b	79.49 a	1394.13 c	433.17 c
**SN**	I	113.60 c	143.23 fg	172.10 f	48.71 ef	7.04 e	54.74 b	2037.70 c	539.85 b
**SN**	II	128.14 c	820.32 c	405.63 d	136.08 d	81.05 a	23.38 de	4410.50 b	440.78 c
**SN**	III	12,813.79 a	3244.17 b	566.60 c	58.90 def	50.30 c	8.31 efg	6040.11 a	361.88 def
**SR**	I	5.51 d	4.28 g	8.13 g	38.82 ef	28.84 d	12.65 defg	2735.02 c	515.60 b
**SR**	II	27.38 d	766.10 cd	620.36 c	84.30 def	2.01 e	26.13 cd	4730.28 ab	808.64 a
**SR**	III	35.16 d	3198.90 b	659.12 c	26.75 f	0.37 e	6.16 fg	4358.81 b	515.63 b
**SV**	I	0.51 d	257.01 ef	180.47 ef	114.48 de	3.58 e	17.05 defg	4550.88 b	415.79 cd
**SV**	II	144.91 c	53.71 g	659.71 c	230.66 c	2.54 e	21.20 def	4807.72 ab	341.04 ef
**SV**	III	150.21 c	44.30 g	619.35 c	229.30 c	0.30 e	15.92 defg	5968.41 a	392.92 cde
**P > F (Model)**		<0.0001	<0.0001	<0.0001	<0.0001	<0.0001	<0.0001	<0.0001	<0.0001
**Significant**		Yes	Yes	Yes	Yes	Yes	Yes	Yes	Yes

Note: Different small letters (a–g) at the same column represent significant differences between the means (*p* < 0.05); *S. retroflexum*—(SR), *S. melanocerasum*—(SM), *S. nigrum*—(SN), *S. villosum*—(SV); Ripening stages I, II, III.

**Table 3:** PCA factors loadings, scores, and eigenvalues for the first two principal components.

Factors	F1	F2
Eigenvalue	5.172	3.516
Variability (%)	36.946	25.117
Cumulative %	36.946	62.062
**Factor loadings**		
Epicatechin	0.541	0.609
*p*-coumaric	−0.099	−0.181
Rutin	0.887	−0.337
Chicoric acid	0.776	−0.462
Ferulic acid	0.816	−0.483
*m*-coumaric	0.930	−0.263
*o*-coumaric	0.569	−0.215
Rosmarinic acid	0.822	−0.142
Myricetin	0.111	0.676
Quercetin	0.262	0.797
Apigenin	−0.574	−0.608
Protocatechuic acid	−0.690	−0.511
Chlorogenic acid	−0.181	−0.416
DPPH radical scavenging activity	−0.177	−0.722
**Factor scores**		
SRI	−1.148	0.110
SRII	−1.721	−1.657
SRIII	−1.329	−1.342
SMI	0.287	4.033
SMII	1.727	0.943
SMIII	6.663	−2.179
SVI	−0.612	1.704
SVII	−1.461	−0.824
SVIII	−2.121	−1.943
SNI	0.706	2.599
SNII	−0.166	−0.378
SNIII	−0.827	−1.065

**Table 4:** Meteorological conditions the experimental period in 2021 and 2022.

Months							
Years	May	June	July	August	September	October	
	Air Temperature °C						Average
**2021**	11.41	19.47	22.51	16.40	11.64	8.13	14.93
**2022**	11.01	17.58	17.99	20.86	11.11	10.07	14.77
**SCN**	13.00	16.30	18.90	17.80	12.90	7.20	14.35
**Rainfall, mm**							**Sum**
**2021**	121.70	40.30	48.40	122.20	29.10	29.80	391.50
**2022**	84.00	77.60	100.50	38.70	26.00	17.70	344.50
**SCN**	53	65	88	77	51	61	395.00

Note: SCN—standard climate normal is the 29-year average from 1991 to 2020. Source: Kaunas Meteorological Station, Lithuania.

## Data Availability

Not applicable.
